# Oxygen desaturation in a transgender man: initial concerns and recommendations regarding the practice of chest binding: a case report

**DOI:** 10.1186/s13256-022-03527-z

**Published:** 2022-09-04

**Authors:** Eugene Kim, Shivali Mukerji, Deen Debryn, Ryan Price, Carl Streed, Ala Nozari

**Affiliations:** 1 Department of Anesthesiology, Boston Medical Center, 750 Albany Street, Power Plant 2R, Boston, MA 02118 USA; 2 Boston University School of Medicine, 72 E Concord St, Boston, MA 02118 USA; 3 Section of General Internal Medicine, Boston University School of Medicine, 72 E Concord St, Boston, MA 02118 USA; 4 Center for Transgender Medicine and Surgery, Boston Medical Center, One Boston Medical Center Pl, Boston, MA 02118 USA

**Keywords:** Ambulatory surgery, Chest binder, Endoscopy, Perioperative guidelines, Transgender medicine

## Abstract

**Background:**

Over 1.4 million US adults identify as transgender when gender identity differs from the sex assigned at birth [1]. Although transgender patients face adverse health outcomes, they remain an understudied population [2]. A 2017 study surveyed 411 practicing clinicians and found that 80% had been involved in treating a transgender patient, but 80.6% had never received training on transgender care [3]. The purpose of this report is to describe prolonged desaturation in one case of a transgender patient who wore a chest binder intraoperatively owing to a lack of preoperative recognition.

**Case presentation:**

A 19-year-old transgender male of African-American descent with anxiety and class 3 obesity presented for an esophagogastroduodenoscopy to evaluate a 2-year history of upper abdominal pain unresponsive to proton pump inhibitor therapy, with a plan for monitored anesthesia care. His medications included sertraline, pantoprazole, zolpidem, ergocalciferol, leuprolide, and testosterone cypionate. Preoperatively, the patient was instructed to remove all clothing and to don a patient gown while in the bathroom. The attending anesthesiologist then conducted the interview and examination in the preoperative holding area. The patient was induced with 250 mg of propofol, and reassuring respirations were noted by capnography. Respirations and oxygen saturation remained stable upon insertion of the endoscope. Four minutes later, the patient’s oxygen saturation rapidly decreased to 50% and end-tidal capnography was lost. The endoscope was removed, and the patient was given 200 mg of propofol and 20 mg succinylcholine. His oxygen saturation recovered to 80% and 100% after 2 and 5 minutes, respectively, of ventilation with 100% inspired oxygen. No further oxygen desaturation was noted throughout the procedure, and the patient was closely monitored for signs of respiratory difficulty during an uneventful postoperative course. After full emergence, it was revealed that the patient had been wearing a chest binder throughout the operative procedure. The patient was counseled on the necessity to communicate the presence of this accessory prior to all future procedures.

**Conclusion:**

In the clinical narrative, a healthy patient was observed to have prolonged oxygen desaturation after induction of anesthesia. Laryngospasm was suspected clinically owing to the sudden absence of end-tidal carbon dioxide. Prolonged oxygen desaturation despite appropriate interventions suggests the contribution of additional factors. We speculate that the presence of a chest binder intraoperatively predisposed the patient to more rapid oxygen desaturation less responsive to typical therapy. A chest binder would introduce mechanical restriction to the patient’s breathing owing to its inherent design to compress. Although the patient was asked to remove all clothing, specific instructions were not provided regarding the removal of a chest binder. The presence of chest binding was also absent in the electronic health record, despite the documented presence of the patient’s preferred gender, hormonal therapy regimen, and medical history. Ultimately, this case reflects the gap between practitioner knowledge and hospital guidelines and the practices of transgender patients. In reviewing existing literature and the potential for atelectasis with external compression, we would consider that patients refrain from chest binding for 12–24 hours before surgical procedures, resume no sooner than 24 hours after ambulation, and participate in diagnostic incentive spirometry pre- and postoperatively.

## Introduction

Over 1.4 million US adults identify as transgender, when gender identity differs from the sex assigned at birth [1]. Although transgender patients face adverse health outcomes, they remain an understudied population [2]. A 2017 study surveyed 411 practicing clinicians and found that 80% had been involved in treating a transgender patient, but 80.6% had never received training on transgender care [3]. The 2015 US Transgender Survey found that 33% of responders had at least one negative experience with clinicians, including 24% who reported having to educate their clinician on transgender concepts in order to receive appropriate care [4].

Of the many transgender-specific practices poorly understood by clinicians, “chest binding”—a practice performed to masculinize appearance by compressing chest tissue with external undergarments—is particularly important for anesthesiologists to recognize. The purpose of this report is to highlight the complications seen in one case of a transgender patient who wore a chest binder intraoperatively. In doing so, we hope to emphasize the importance of recognizing chest binding and of creating a welcoming perioperative environment for transgender patients who may feel uncomfortable discussing this practice.

Written Health Insurance Portability and Accountability Act authorization was obtained from the patient described.

## Case description

A 19-year-old transgender man of African-American descent with posttraumatic stress disorder, anxiety, panic disorder, and bipolar disorder presented for an esophagogastroduodenoscopy (EGD) for evaluation of left upper-quadrant abdominal pain. He weighed 124 kg, and his body mass index (BMI) was 46.9 kg/m [2]. He reported a history of snoring and did not endorse other comorbidities. The patient’s abdominal pain had been present for 2 years and had not responded to proton pump inhibitor therapy. His medications included sertraline, pantoprazole, zolpidem, and ergocalciferol, as well as leuprolide and testosterone cypionate as gender-affirming hormone therapy.

On the day of the procedure, the patient was instructed to step into a changing room, remove all clothing, and don a patient gown. The patient responded affirmatively when asked if he had removed all clothing and undergarments.

**Fig. 1:**
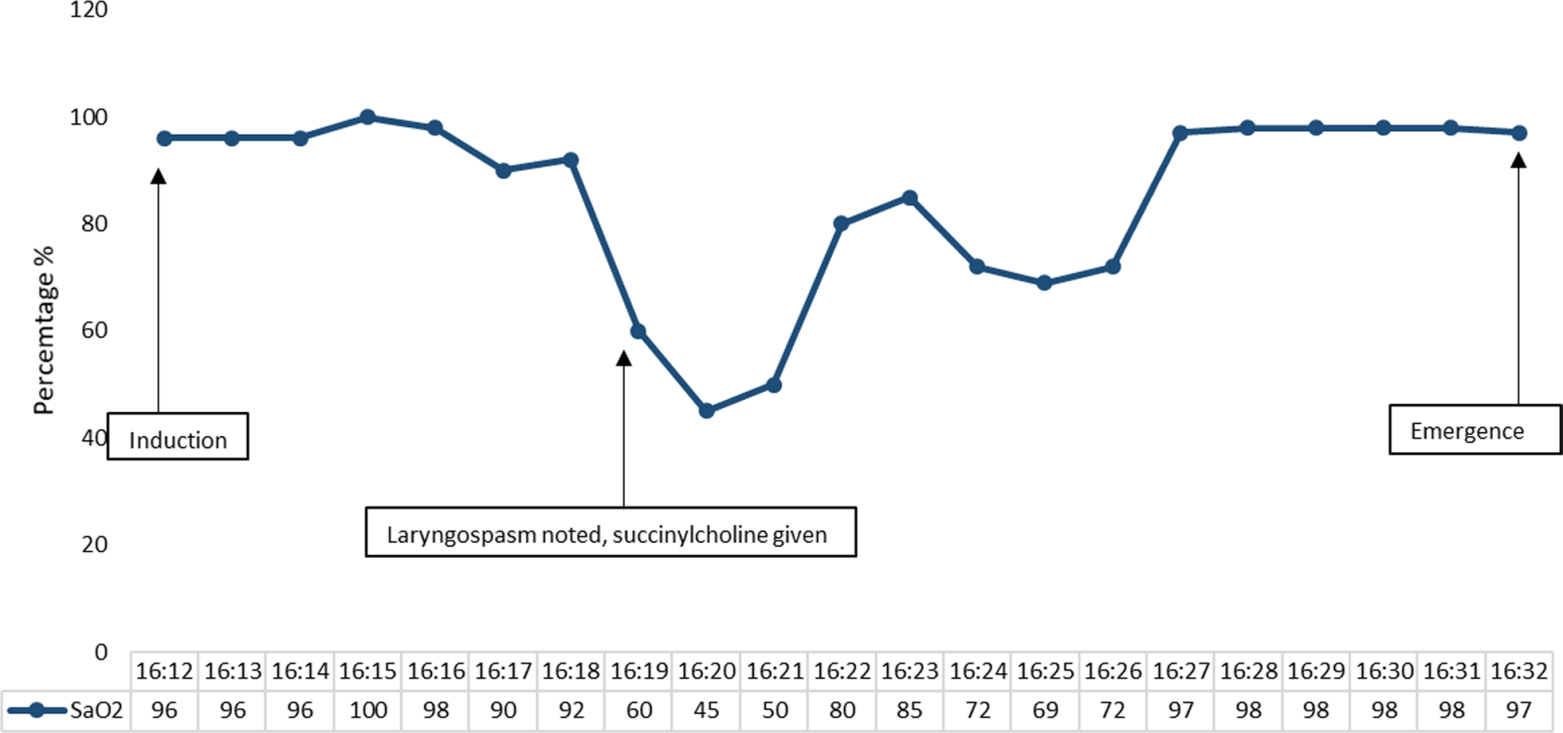
Changes to the patient’s oxygen saturation (SaO_2_) from induction to emergence

In the procedure room, monitors for pulse oximetry, capnography, and blood pressure were placed on the patient. He was given 35% inspired oxygen via nasal cannula. Anesthesia was induced with 250 mg of propofol, and reassuring respirations were noted by capnography. Upon initial insertion of the endoscope, respirations and oxygen saturation (SaO_2_) remained stable. Four minutes later, the patient’s SaO_2_ decreased rapidly to 50% and end-tidal respirations were lost. The endoscope was removed, the patient was given 200 mg of propofol and 20 mg succinylcholine and was ventilated by bag mask. His SaO_2_ recovered to 80% after 2 minutes and to 100% after 5 minutes of ventilation with 100% inspired oxygen. Changes in SaO_2_ are detailed in Fig. 1. There were no ST segment changes or bradycardia noted on electrocardiogram during the procedure.

Endoscopy was resumed with no airway device and without further oxygen desaturation. Upon conclusion of the procedure, the patient was closely monitored for signs of respiratory difficulty in the post-anesthesia care unit. Auscultation of the patient’s lung fields were unremarkable, and his postoperative course was uneventful.

**Fig. 2:**
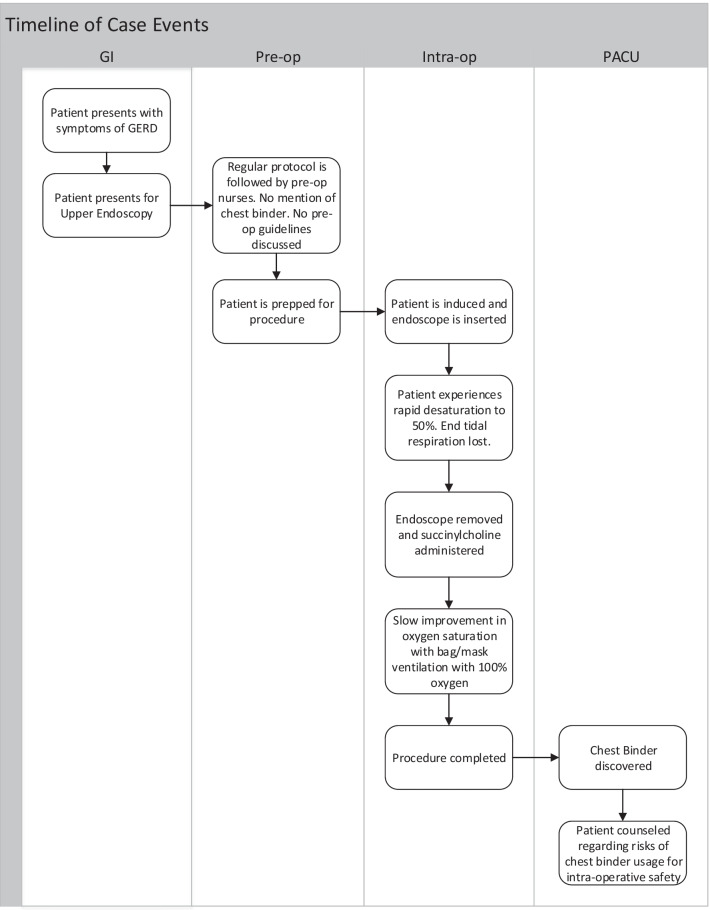
Timeline of case events

After full emergence from anesthesia, the event was discussed with the patient. At this time, it was revealed that the patient was still wearing a chest binder. The patient was counseled on the necessity to communicate the presence of this accessory to clinicians prior to all future procedures. Figure 2 summarizes the case events.

## Discussion

In the clinical narrative, a healthy patient was observed to have prolonged oxygen desaturation. As suggested in current literature, the patient in this case had several risk factors for desaturation while sedated, including his young age, obesity, and potential sleep apnea [5–7]. Clinically, laryngospasm was suspected owing to the sudden absence of end-tidal carbon-dioxide detection. However, the patient’s prolonged oxygen desaturation despite appropriate interventions of succinylcholine and positive pressure ventilation of 100% inspired oxygen suggests contribution of additional factors. We speculate that the presence of a chest binder intraoperatively can result in a reduced functional residual capacity (FRC) predisposing the patient to more rapid oxygen desaturation that is less responsive to typical therapy. Indeed, a previous study among a cohort of transgender men showed an association between regular chest binding and overall reduction in lung volume [8]. A decrease in FRC would also prevent effective recruitment of alveoli and thus prolong shunting and time to recovery in the setting of oxygen desaturation. Moreover, a chest binder can impede effective alveolar recruitment by introducing mechanical restriction to the patient’s breathing due to its inherent design to compress.

While chest binding may have negatively affected the patient’s time to reoxygenation, the presence of testosterone hormone therapy may also have been a risk factor for oxygen desaturation. A recent systematic review concluded that exogenous testosterone administration in transgender men was associated with modest increases in BMI [9]. An increase in BMI leads to an increase in airway soft tissue, the likelihood of obstructive sleep apnea (OSA), and the possibility of a difficult airway. Although research in this area is lacking, a recent literature review investigating the 40-year history of testosterone therapy in cisgender men with OSA concluded that testosterone plays a small role in induction or exacerbation of OSA symptoms, and that clinicians should exercise caution when prescribing testosterone to patients with OSA [10].

In the case presented above, routine protocols were followed regarding clothing transition for perioperative care. Although the patient was asked to remove all clothing, specific instructions were not provided regarding removal of a chest binder. The presence of chest binding was also absent in the electronic health record, despite the documented presence of the patient’s preferred gender, hormonal therapy regimen, and medical history. Given the absence of documentation, perioperative nursing staff may not have deemed it necessary to inquire about the presence of chest binding material. This mistake reflects the gap between practitioner knowledge, hospital guidelines, and the practices of transgender patients.

Several medical organizations have created guides detailing chest binding use for both patients and clinicians. These include “full-length binders,” which extend from the neckline to the waist, and “half-length binders,” which cover only the ribs [12]. This case report highlights the importance of using clear unequivocal terms when directing preoperative direction for clothing transition [13].

There are no existing recommendations for patients who engage in chest binding for the perioperative period. However, in reviewing existing literature and the potential for atelectasis with external compression, we would consider that patients refrain from chest binding for 12–24 hours before surgical procedures, and resume no sooner than 24 hours after ambulation. We would also consider that perioperative teams have patients participate with incentive spirometry before and after anesthesia. Ultimately, proper and progressive perioperative guidelines will be needed to ensure transgender patients’ safety during surgical procedures without compromising their right to privacy and comfort, given the well-documented discomfort that transgender patients experience in healthcare settings.

## Conclusion

In the clinical narrative, a healthy patient was observed to have prolonged oxygen desaturation after induction of anesthesia. Laryngospasm was suspected clinically owing to the sudden absence of end-tidal carbon dioxide. Prolonged oxygen desaturation despite appropriate interventions suggests the contribution of additional factors. We speculate that the presence of a chest binder intraoperatively predisposed the patient to more rapid oxygen desaturation less responsive to typical therapy. While the patient does possess multiple comorbidities that may have extended the duration of the desaturation, we suggest that the presence of a chest binder may have been one more. A chest binder would introduce mechanical restriction to the patient’s breathing owing to its inherent design to compress. Although the patient was asked to remove all clothing, specific instructions were not provided regarding the removal of a chest binder. The presence of chest binding was also absent in the electronic health record, despite the documented presence of the patient’s preferred gender, hormonal therapy regimen, and medical history. Ultimately, this case reflects a gap between practitioner knowledge along with hospital guidelines and the practices of transgender patients.

## Data Availability

Data sharing not applicable—no new data generated

## Abbreviations

BMI: Body mass index
EGD: Esophagogastroduodenoscopy
FRC: Functional residual capacity
GERD: Gastroesophageal reflux disease
OSA: Obstructive sleep apnea
SaO_2_: Oxygen saturation
